# Neurocognitive dysfunctions in childhood-onset schizophrenia: A systematic review

**DOI:** 10.1016/j.scog.2024.100342

**Published:** 2025-01-05

**Authors:** A. Armita, J. Guivarch, E. Dor, G. Laure, R. Zeghari, M. Gindt, S. Thümmler, F. Askenazy, A. Fernandez

**Affiliations:** a University Department of Child and Adolescent Psychiatry, Children's Hospitals of NICE CHU-Lenval, Nice, France; b Université Côte d'Azur, CoBTek, France; c Faculty of Medicine, Aix-Marseille University, Marseille, France; d Centre de Référence pour les Maladies Rares à expression psychiatrique (PsyRare), Nice, France; e Department of Child Psychiatry, AP-HM, Marseille, France; f Institut de Neurosciences de la Timone, UMR 7289, CNRS, Aix-Marseille University, Marseille, France

**Keywords:** Childhood-onset schizophrenia, Neurocognitive dysfunction, Neuropsychology, IQ, Attention, Memory, Executive function

## Abstract

**Objective:**

To conduct a systematic review of neurocognitive dysfunctions in patients with childhood-onset schizophrenia (COS), a neuropsychiatric disorder that occurs before age 13 and is rarer and more severe than adult-onset schizophrenia.

**Method:**

A search was made in the PubMed database. Sixty-seven studies (out of 543) which analyzed Intellectual Quotient (IQ), attentional, memory and executive functions were selected by two independent researchers. Study's appraisal was done according to the Mixed Methods Appraisal Tool (MMAT). This systematic review was registered on PROSPERO (CRD42024548945).

**Result:**

COS shows neurocognitive dysfunction in IQ with mean scores ranging from one to two standard deviation lower than normative data. Attentional deficits are observed with longer reaction time, more errors of omission and commission and slower processing speed than controls. In addition, working memory and executive functions, such as planification and flexibility are impaired. COS exhibit significantly more neurocognitive deficits than adolescent and adult-onset forms and display deterioration in intellectual functioning between premorbid period and after onset of psychosis.

**Conclusion:**

COS is characterized by major cognitive impairments, both before the onset of the disease and throughout its course. As in adult-onset schizophrenia, generalized cognitive impairment is found without the emergence of a specific profile, providing further support for the continuum hypothesis between early-onset and adult-onset schizophrenia. Collaborative research on a larger scale (including meta-analyses) and using complementary approaches (dimensional and multimodal) is needed to gain a better understanding of the cognitive impact of COS and pave the way for more precise and targeted cognitive remediation.

## Highlights

• Children with COS show IQ scores 1-2 SD below average, with significant decline from premorbid to post-onset psychosis
• COS shows greater impairment and a poorer prognosis than adult forms with symptom severity closely tied to lower IQ
• As in adult-onset schizophrenia, no specific deficits have been identified, but rather global cognitive impairment

## 1 Introduction

Childhood-Onset Schizophrenia (COS) is a rare, severe and neurodevelopmental neuropsychiatric disorder that begins before the age of 13 (Driver et al., 2020). COS is not listed in the DSM-5-TR (APA, 2022) and is considered under the axis of Adult-Onset Schizophrenia (AOS). It is characterized, as in adults, by the appearance of positive symptoms (i.e. delusions, hallucinations, and disorganized speech/behavior), negative symptoms (i.e. social withdrawal and avolition) and cognitive impairments (APA, 2022). However, patients with COS present more severe clinical symptoms and a worse prognosis (Coulon et al., 2020) compared to those with AOS.

Although little specific etiological work has been done on patients with this early form, the neurodevelopmental model is now widely accepted (Rapoport and Gogtay, 2011; Pontillo et al., 2021). Moreover, premorbid neurodevelopmental abnormalities (including cognitive impairment) and overlaps with other neurodevelopmental disorders have been well described in this population (Driver et al., 2020; Giannitelli, et al., 2020; Kumra et al., 2000, Kumra et al., 2001a; Remschmidt, 2002; McKenna et al., 1994). These are mainly Autism Spectrum Disorder (ASD) and Attention Deficit Hyperactivity Disorder (ADHD; Ross et al., 2006; Rapoport et al., 2009; Fernandez et al., 2021).

As COS is a rare, severe, neurodevelopmental neuropsychiatric disorder, frequently linked to other neurodevelopmental/psychiatric disorders, it poses a diagnostic (Gochman et al., 2011) and therapeutic challenge with severe prognostic outcomes.

Cognitive impairment in AOS is known to be a major cause for the severity of the prognostic and for the functional impairment in patients (Martinez-Aran and Vieta, 2015). However, according to the commonly accepted hypothesis of a continuum between COS and AOS (Driver et al., 2020), we should find in the COS population the same global and unspecific cognitive impairments as those described in AOS (Martinez-Aran and Vieta, 2015), with a degree of severity inversely proportional to age of onset (Coulon et al., 2020).

To our knowledge, a systematic review of the literature has never been carried out with the aim of identifying specific cognitive impairment in COS, to the exclusion of that presented in AOS. Yet cognitive impairment specific to COS would enable the implementation of early, targeted cognitive remediation programs in the hope of improving the prognosis outcomes.

We therefore sought to explore the literature in order to identify cognitive impairments specifically associated with the COS population.

## 2 Method

We conducted a systematic review of the MEDLINE database accessible via the PubMed search engine (www.ncbi.nlm.nih.gov/pubmed/) with the following keywords (“childhood schizophrenia” or “childhood-onset schizophrenia” or “childhood-onset psychosis” or “very- early onset schizophrenia”) and (“neuropsychology” or “neuropsychologic” or “cognition” or “cognitive” or “neuropsychological” or “memory” or “learning” or “recognition” or “attention” or “focus” or “executive” or “flexibility” or “intellectual” or “inhibition” or “IQ” or “speed” or “verbal skills” or “perceptual skills” or “crystallized knowledge” or “wais” or “wppsi” or “wisc” or “Wechsler”). Our search terms were not restricted by language or publication date and were manually reviewed. ChatGPT was used for minimal translation, data extraction and review verification. We considered all cognitive disorders occurring in patients with COS (age of onset before 13 years). We excluded from this review all articles dealing with molecular aspects (including genetics) and all animal models of COS. We also excluded Unspecified Schizophrenia Spectrum and Other Psychotic Disorder of children psychosis of Unspecified Schizophrenia Spectrum and Other Psychotic Disorder. Diagnostic tools for schizophrenia (DSM or ICD version), neuropsychological tools (WISC, WAIS, WASI version) and type of tools for cognitive processes will be assessed for case reports, case series, reviews and meta-analysis. Two reviewers will be involved for quality assessment. The Mixed Methods Appraisal Tool (MMAT) by Hong et al. (version 2018) was used for the appraisal stage of the reviewing process for qualitative research, randomized controlled trials, non-randomized studies, quantitative descriptive studies, and mixed methods studies. We have also registered this systematic review on PROSPERO, its ID is CRD42024548945.

**Fig. 1:**
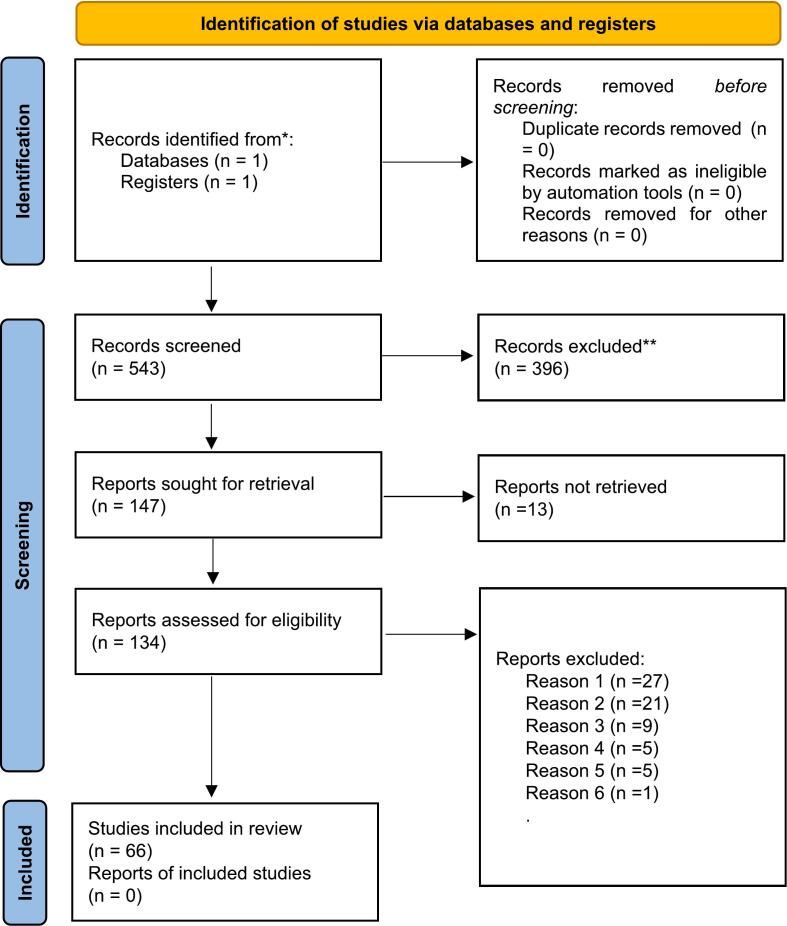
PRISMA 2020 flow diagram for new systematic reviews which included searches of databases and registers only. Reason 1: Not COS. Reason 2: No neurocognitive or neuropsychological data available or not WISC (old intelligence scale, such as Wechsler-Bellevue, Standford-Binet or non-verbal intelligence scale). Reason 3: No sufficient data. Reason 4: Neurocognitive and neuropsychological data for children or parents. Reason 5: Merged cohort (ASD and COS or COS and shizoaffective disorder). Reason 6: No clear method (no tools specified).

The article review process, including selection and exclusion, is summarized in a PRISMA diagram (Fig. 1).

From: Page MJ, McKenzie JE, Bossuyt PM, Boutron I, Hoffmann TC, Mulrow CD, et al. The PRISMA 2020 statement: an updated guideline for reporting systematic reviews. BMJ 2021;372:n71. doi: https://doi.org/10.1136/bmj.n71.

## 3 Results

The selection took place on June 4th, 2024. At that date, 66 articles (1958 to 2024) out of 543 (1947 to 2024) met the inclusion criteria.

For more readability, we have grouped the obtained results by neuropsychological substrates: Intellectual Quotient (IQ), memory, executive function and verbal and learning disabilities.

### 3.1 Neuropsychological substrate of intellectual quotient (IQ)

#### 3.1.1 Full Scale Intellectual Quotient (FSIQ)

FSIQ scores measured by the various studies average between 63.3 and 105, with standard deviations between 7.1 and 25.4. For the most part, studies reveal mean FSIQ scores ranging from 1 to 2 standard deviation lower than normative data (norm set at 100 with a standard deviation of 15; Caplan et al., 1992; Kumra et al., 2000; Remschmidt, 2002; Kumra et al., 2001a; Biswas et al., 2006; Fernandez et al., 2021). Furthermore, 18 to 40 % of COS patients are estimated to show FSIQ under the norms (Pollack, 1958) and approximately 15 % of them also meet the criteria for intellectual disability (Fourneret et al., 2013). Table 1 below reports the mean FSIQ scores, standard deviation for COS patients with other characteristics (mean age of onset, mean age of assessment, diagnostic tool, exclusion criteria, FSIQ scale used, when available).

**Table 1:** Full Scale IQ scores, measures and characteristics for COS and comparative group.

Authors	*M*	*SD*	*N*	COS					Comparison group(s)			Scale
				Mean age of onset in yrs ±SD	Mean age in yrs ± SD	M/F ratio	Diagnostic tools	IQ exclusion criteria	*M*	*SD*	*N*	
Wechsler and Jaros (1965)	93. 8^a^	7.7	100	Diag. last 7 yrs	Range 8–11.9	100M	NA	IQ < 80 IQ > 120	94.0^b^	8.0	100	WISC^c^
Czudner and Marshall (1967)	63.3	NA	17	NA	Range 10–16	17/3^d^	NA	NA	NC	NC	NC	WISC^c^
Goldfarb et al. (1969)		NA	26	NA	6.7	17/9	NA	NA	NC	NC	NC	WISC^c^
	74.8^e^		18		6.8	14/4						
	80.9											
	81.8											
	83.2											
	91.1^f^		8		6.6	3/5						
	94.1											
	96.9											
	101.4											
Walker and Birch (1974)	84.95	9.86	20	NA	10	120M	NA	NA	NC	NC	NC	WISC^c^
	80.90	15.07	20		11							
	78.05	11.67	20		12							
	75.65	13.60	20		13							
	81.95	17.42	20		14							
	84.30	16.09	20		15							
	80.97^g^	Range 46–117	120									
	71^h^		63									
	93^i^		57									
Walker and Bortner (1975)	86.50^j^	21.67	25	NA	9.5 ± 0.87	48M	DSM III	NA	Range 30–118^m^	NA	104	WISC^c^ Stanford-Binet Form L-M
		Range 40–123										
	79.63^k^	18.68	23		11.8 ± 0.57							
		40–108										
	82.21^l^	20.23	48		10.6 ± 1.38							
		40–123										
Fish (1977)^n^	65^o^		1	3	10	1M	NA	NA	NC	NC	NC	WISC^c^ WAIS^c^
	81	NA			18							
	84		1	6	10	1F						
Caplan and Walker (1979)	71.9	NA	30	NA	13y4m	NA	NA	NA	79.7^p^	NA	30	WISC^c^
Inayatulla and Cantor (1980)^n^	72^q^ 79	NA	1	10yrs2m	11y6m 11y10m	1M	DSM III	NA	NC	NC	NC	WISC-R
Carter et al. (1982)	84.3	16.75	15	NA	9.2	12/3	NA	NA	89.0^r^	16.19	18	WISC^c^
Watkins et al. (1988)	86.3	Range 78–104	18	Prior or at 10 yrs	Range 9–11.11	13/5	DSM-III	IQ < 70	NC	NC	NC	WISC-R Stanford Binet test (N = 1)
Russell et al. (1989)	85.9^s^	Range <69–>110	33	NA	11.1^u^	24/9	NA	NA	NC	NC	NC	WISC-R^w^
					Range 5–15							
	88.5^t^	16.0	24	NA	9.96 ± 1.63	15/9	NA	NA	NC	NC	NC	
		65–125										
	94	10.5	35	6.9	9.54 ± 2.07^v^	24/11	DSM III	IQ < 70	NC	NC	NC	
		76–114		Range 3–11								
Strandburg et al. (1990)	96	8.1	13	Range 7–9	11.2 ± 1.5	8/5	DSM III	NA	NA	NA	NA	WISC^c^
Caplan et al. (1990a)	90	12.37	31	NA	10.3 ± 1.51	25/6	DSM III	NA	NC	NC	NC	WISC-R
Caplan et al. (1990b)	90	10.7	29	NA	10.2 ± 1.6	23/6	DSM III	NA	115^x^	11.7	29	WISC-R
Caplan et al. (1992)	90	12.37	31	6.8 ± 1.98	10.3 ± 3.06	25/6	DSM III	NA	90^y^ 113^b^	23.07 15.09	27 58	WISC-R
Asarnow et al. (1994)	87.0	9.6	24	NA	10.5 ± 1.9	34/13^z^	DSM-III-R	IQ < 70	91.4^aa^	16.1	23	WISC-R
Russell (1994)	96.4	10.5	35	6.9	At diag. M = 9.45 Range 4.75–13.33	24/11	DSM III	IQ < 70	NC	NC	NC	WISC-R
Alaghband-Rad et al. (1995)	87.7^ab^ 83.7^ac^	25.4 17.3	7	Psychotic symptoms prior or by age 12	14.3 ± 1.8^ad^	15/8^ae^	DSM-II/-R	NA	NC	NC	NC	WISC^af^
Gartner et al. (1997)^n^	105^ag^ 82 91	NA	1	NA	12	1M	DSM-IV	NA	NC	NC	NC	WISC-R WISC-III
Kumra et al. (1998)	74.83^ah^ 84.60^ai^	20.11 16.58	29 15	10.31 ± 2.00	14.03 ± 2.16	14/15	DSM-III-R	Premorbid IQ < 70	96.11^aj^ 85.29^ak^	9.07 7.71	19 14	WISC-R
Nicolson et al. (1999)	70.2^al^ 75.4	5.6 20.6	5 42	10.3 ± 1.8	14.3 ± 2.3	28/19	DSM-III-R	Premorbid IQ < 70	NC	NC	NC	WISC-III
Abu-Akel et al. (2000)	89.96^am^ 90.29^an^ 89.60^ao^	12.72 15.96 8.19	32 17 15	NA	10.34 ± 1.56 10.37 ± 1.67 10.32 ± 1.48	27/5 13/4 14/1	DSM-III DSM-IV	NA	106.29 ^b^	12.44	34	WISC-R
Kumra et al. (2000)	84.9	15.1	17	10.7 ± 1.6	14.4 ± 1.8	12/5	DSM-III-R/DSM-IV	Premorbid IQ < 70	87.9^ap^	9.7	21	WISC-R
Thompson et al. (2001)	70.4	12.9	12	Psychotic symptoms by age 12	13.9 ± 0.8	6/6	DSM-III-R	NA	76^aq^	10	10	NA
Ballmaier et al. (2004)	95.50	13.03	12	NA	11.4 ± 3.0	6/6	DSM-IV	NA	119.80 ^b^	14.54	15	WISC-R
Gochman et al. (2005)	90.0^ar^ 80.0^as^ 73.1^at^ 76.4^au^ 76.1^av^ 77.2^aw^ 70.6^ax^	20.0 11.1 19.0 18.6 16.5 15.0 7.1	18 13 70 34 33 16 6	11.6 ± 1.6	8.6 ± 2.0 11.6 ± 1.6 14.3 ± 2.4 16.9 ± 2.6 18.7 ± 2.2 22.1 ± 2.8 24.3 ± 1.5	15/9 10/6 42/28 20/14 18/15 12/4 4/2	DSM-III-R/IV	IQ < 70	NC	NC	NC	WISC-R WISC-III
Biswas et al. (2006)	88.33	11.13	15	12.25 ± 1.16	18.52 ± 3.98	7/8	ICD-10 DCR	<70	99.15^ay^ 109.60^az^	13.78 9.96	20 20	WAIS-R, MISIC
Vidal et al. (2006)	68.0^ba^ 70.2^bb^	17.5 14.6	12	Onset by 12 yrs	14.1 ± 2.7	6/6	DSM-III-R	NA	77.9^bc^ 77.0^bd^	12.8 11.1	9	WISC^c^ WAIS^c^
Stenstrøm et al. (2008)^n^	105	NA	1	NA	7	1M	CIM10	NA	NC	NC	NC	WISC III
Rapoport et al. (2009)	79.33^be^ 74.10	15.50 18.84	28 73	9.76 ± 2.18 10.35 ± 1.94	NA	71 % M 52 % M	DSM-III/IV/IV-TR	NA	NC	NC	NC	NA
Borofsky et al. (2010)	95.36	11.32	14	NA	13.34 ± 2.14	7/7	K-SADS-PL	NA	104.15 ^b^	7.87	14	NA
David et al. (2011)	83.3^bf^ 71.2^bg^	21.0 17.4	24 94	10.7 ± 1.59 9.7 ± 2.1	15.1 ± 2.62^r^ 13.23 ± 2.79^s^	67/50	NA	IQ= or <70	NC	NC	NC	NA
Greenstein et al. (2014)	71.20	8.25	85	9.92 ± 2.06	13.21 ± 2.42	56 % M	DSM-IV	FSIQ <70	82.00^bh^	16.01	53	WISC^c^ WASI
Ordóñez et al. (2016)	79.02^bi^ 74.66^bj^	16.20 16.33	53 38	9.51 ± 2.28^bk^ 10.29 ± 1.63	NA	53/38	DSM-III-R/DSM-IV	Premorbid IQ < 70	NC	NC	NC	WISC-R WISC-III WAIS-R WASI WASI II
Aneja et al. (2018)^n^	90	NA	1	12	14	1M	NA	NA	NC	NC	NC	NA
Craddock et al. (2018)	80.16	16.99	114	9.90 ± 2.03^bl^	13.32 ± 2.68	65/60 Data for *N* = 125	DSM-III R/DSM-IV	Premorbid IQ < 70	NC	NC	NC	WISC III WISC-R
Coulon et al. (2020)	83.25	12.97	22	9.55 ± 2.5	24.1 ± 6.2	14/8	DSM IV-TR	NA	82.65^bm^ 84.36^bb^	16.08^bm^ 15.24^bb^	154 551	WAIS-IV^bn^
Fernandez et al. (2021)	70.26	18.09	20	8.90 ± 2.34	13,60 ± 3.66	15/5	K-SADS-PL - DSM-5	IQ < 40	NC	NC	NC	WISC-V WAIS-IV
Adhikari et al. (2022)^n^	70	NA	1	11	12	1F	NA	NA	NC	NC	NC	NA

NA: data not available; NC: no comparison group.

a Study limited to COS children with an IQ greater than or equal to 80 (Wechsler and Jaros, 1965).

b Mean score and standard deviation for the healthy control comparison group.

c WISC or WAIS version not specified.

d Ratio from overall cohort (*N* = 20).

e Mean score at admission, 1st, 2nd and 3rd year after admission, mean age and ratio for schizophrenic children with positive neurological findings (organic).

f Mean score at admission, 1st, 2nd and 3rd year after admission, mean age and ratio for schizophrenic children with no positive neurological findings (nonorganic).

g Mean score and range for overall cohort (*N* = 120).

h Mean FSIQ and N for COS patients with IQ < 80.

i Mean FSIQ and N for COS patients with IQ/>80.

j Mean score, standard deviation, range, N and mean age for group age 8–10.

k Mean score, standard deviation, range, N and mean age for group age 10–12.

l Mean score, standard deviation, range, N and mean age for overall cohort.

m *N* = 104 cerebral-palsied (brain-injured) children with an age span of 5 to 21.

n Case study *N* = 1.

o Full scale IQ at 10 years old FSIQ_10_ = 65; at 18 years old FSIQ_18_ = 81 for same male patient; FSIQ_10_ = 84 for female patient.

p Comparison group composed of children with the diagnosis that were less severe and nonpsychotic in nature, such as behavior disorder, unsocialized aggression, and neurosis.

q Mean scores for same patient at two separate occasions.

r 18 patients hospitalized for psychiatric conditions other than schizophrenia: overanxious reaction of childhood (*N* = 10), unsocialized aggressive reaction of childhood or dyssocial behavior (*N* = 4), adjustment reaction of childhood or adolescence (*N* = 3), and depressive reaction of childhood (*N* = 1) (Carter et al., 1982).

s Mean score, standard deviation, mean age and ratio for Kolvin, et al., 1971 presented in Russell et al. (1989).

t Mean score, standard deviation, mean age and ratio for Green, et al., 1984 presented in Russell et al. (1989).

u Mean age and ratio for *N* = 33, overall cohort.

v Ratio for *N* = 35, overall cohort.

w Scale only available for Russell et al. (1989).

x Mean score and standard deviation for the control comparison group matched in IQ, age and sex.

y Mean score and standard deviation for complex partial seizures (epilepsy) patients.

z Ratio for *N* = 47, overall cohort, not only COS who undergone FSIQ testing.

aa Mean score and standard deviation for subjects with ASD.

ab “Prepsychotic” mean and standard deviation for COS subjects.

ac “Postpsychotic” mean and standard deviation for COS subjects.

ad Mean age and ratio for *N* = 23, overall cohort, not only COS with available FSIQ data.

ae Ratio for overall cohort (N = 23).

af WISC version not specified and only Verbal subscale mentioned.

ag Patient's FSIQ score in Middle of Grade 2 WISC-R, FSIQ score in Fall of Grade 6 WISC-III and FSIQ score in Spring of Grade 7, Shortly After Hospital Admission.

ah Average score for all COS subjects.

ai Mean score for COS subjects with IQ > 70.

aj Mean score and standard deviation for the ADHD comparison group.

ak Mean score and standard deviation for the Multidimensionally Impaired Disorder (MDI) comparison group.

al Mean score, standard deviation and N for COS with cytogenetic abnormalities.

am Mean score, standard deviation and ratio for all COS.

an Mean score, standard deviation and ratio for COS on Neuroleptics.

ao Mean score, standard deviation and ratio for COS off Neuroleptics.

ap Mean score and standard deviation for the Psychotic Disorder Not Otherwise Specified (PD-NOS) comparison group.

aq Mean score and standard deviation for the healthy control comparison group matched in IQ, age, gender and demographics.

ar Mean score and standard deviation of COS patients pre-onset.

as Mean score and standard deviation of COS patients post-onset.

at Mean score and standard deviation of COS patients at NIH admission.

au Mean score and standard deviation of COS patients at 2 years follow-up.

av Mean score and standard deviation of COS patients at 4 years follow-up.

aw Mean score and standard deviation of COS patients at 6 years follow-up.

ax Mean score and standard deviation of COS patients at 8+ years follow-up.

ay Mean score and standard deviation of AdOS: Subjects with adolescent schizophrenia.

az Mean score and standard deviation of AOS: Adult schizophrenia subjects.

ba Mean score and standard deviation for COS patients at baseline.

bb Mean score and standard deviation for COS patients at follow up (5 years).

bc Mean score and standard deviation for PNOS patients at baseline.

bd Mean score and standard deviation for PNOS patients at follow up (5 years).

be Mean score, standard deviation and age of onset for COS patients with PDD (Pervasive Developmental Disorder).

bf Mean score and standard deviation of COS patients without visual hallucination.

bg Mean score and standard deviation for COS patients with visual hallucinations.

bh Mean score and standard deviation for patients with an “*alternative diagnosis*”.

bi Mean score and standard deviation for male COS patients.

bj Mean score and standard deviation for female COS patients.

bk Mean age of onset for overall cohort (*N* = 72 males; *N* = 61 females), not only patients who undergone FSIQ testing.

bl Age of onset for *N* = 124 and mean age for *N* = 121 COS patients.

bm Mean score and standard deviation of EOS: Subjects with Early-Onset Schizophrenia after 13 years old.

bn WAIS-IV 7 subtests according to authors.

For most studies reviewed, inclusion criteria threshold was set at FSIQ value above 70, with exceptions (Wechsler and Jaros, 1965; Thompson et al., 2001; Vidal et al., 2006; Greenstein et al., 2014; Fernandez et al., 2021). For those who set a lower exclusion threshold, results show FSIQ mean score averages close to intellectual disability threshold (Thompson et al., 2001; Vidal et al., 2006; Rapoport et al., 2009). In particular, Fernandez and colleagues observed intellectual disability in 10 patients (50 %), defined by a FSIQ of less than −2 standard deviations (i.e. <70) from the mean. Mean FSIQ for all subjects was 70.26 ± 18.09 (Fernandez et al., 2021). However, some studies found FSIQ scores within the norms (Watkins et al., 1988; Russell et al., 1989; Caplan et al., 1990a; Caplan et al., 1990b; Caplan et al., 1992; Russell, 1994; Ballmaier et al., 2004; Biswas et al., 2006 Stenstrøm et al., 2008; Borofsky et al., 2010; Aneja et al., 2018) although standard deviations scores indicate a wide disparity of scores within each cohort. Pollack (1958) observed frequency scores falling within the normal range, 90 or above, varied between 10 and 23 % in COS patients whereas the incidence of children with FSIQs below 69 ranged from 30 to 40 %. Upon averaging three studies, 20 % of COS children fell within the normal range, 44 % were of borderline or dull normal intelligence, and 34 % were “subnormal”. According to the authors, these results demonstrate the marked overlap in intellectual functioning between COS and children diagnosed with intellectual disability, only observable for testable children. Wechsler and Jaros (1965) looked for indicators of discrimination in COS patients' profile for WISC (no version specified). One discriminating sign was the presence of three subtests deviating by at least three calibrated points from the mean, later refuted by Kissel (1966) as discovered in other diagnostic groups, such as non-schizophrenic emotionally disturbed children.

As regards to comparison between age groups, COS' FSIQ scores were significantly lower than adolescent (AdOS) and adult-onset schizophrenia (AOS) patients. Respectively, *M* = 99.15, *SD* = 13.78 for adolescent subjects, *M* = 109.60, *SD* = 9.96 for adult subjects and *M* = 88.33, *SD* = 11.13 for COS. These statistical analyses reveal significantly different results *F*_(2)_ = 14.01 *p* < .001 between the three groups (Biswas et al., 2006). On the other hand, Coulon et al. (2020) found no significant difference in FSIQ scores between COS, adolescent and adult schizophrenia groups (*p* = .523 three groups, *p* = .699 between COS and AdOS FSIQ and *p* = .913 between COS and AOS). There was also no difference in premorbid IQ scores between the three groups. Furthermore, Rapoport et al. (2009) showed no significant difference between COS' FSIQ mean scores with (*M* = 79.33 *SD* = 15.50) and without ASD (*M* = 74.10; *SD* = 18.84; *t*_(94__–__68)_ = 1.06, *p* = .29). However, when compared with ASD children, similar FSIQ mean scores are found for COS children (*M* = 87.0, *SD* = 9.6) and ASD children (*M* = 91.4, *SD* = 16.1). Both scores falling within the low normal range (Asarnow et al., 1994). No difference was observed in cognitive performance between COS with and without PDD diagnostic (Sporn et al., 2004). Kumra et al. (2000) found that both COS and PD-NOS patients exhibited cognitive deficits, with neuropsychological tests results falling 1 to 2 standard deviations below the norm across various cognitive domains. Only 8 % of patients (3 out of 38; two PD-NOS, one COS) had a mean cognitive functioning score (FSIQ) above 90 for six domains of FSIQ. The PD-NOS and COS groups showed similar deficits in both nature and severity, although COS patients had fewer deficits on the WISC-R Code and Symbol subtests compared to the PD-NOS group.

Conversely, Caplan et al. (1992) reported no significant differences in mean WISC-R scores and factor scores of epileptic and COS patients (*M*_epilepsy_ = 90, *SD* = 23.07; *M*_COS_ = 90, *SD* = 12.37). Both means were more than one standard deviation less than healthy comparison group (*M*_*Healthy*_ = 113, *SD* = 15.09). Pollack (1958) found that the percentage of children with behavioral disorders scoring above 90 on FSIQ was twice as high compared to COS group. Conversely, COS children were three times as likely to score below 69. Combined data revealed a significantly greater proportion of COS children scoring below the FSIQ threshold of 70, indicative of intellectual disability, compared to the behavioral disorder group. Finally, Walker and Birch (1974) mentioned the resemblance of higher FSIQ COS to “emotionally disturbed children” and lower mean FSIQ scores for COS compared to “brain damaged children”.

#### 3.1.2 Verbal Comprehension Factor (VC) and Verbal IQ (VIQ)

Verbal IQ (VIQ) values ranged on average from 52 to 106 with standard deviations from 7.7 to 29.1 depending on the study. These VIQ and Verbal Comprehension (VC) values are 1 to 2 standard deviations lower than the normative data (Walker and Birch, 1974; Nicolson et al., 1999; Remschmidt, 2002; Bailly and de Chouly de Lenclave, 2004; Inayatulla and Cantor, 1980; Ordóñez et al., 2016; etc.) although some studies show scores within the norms (Kumra et al., 2000; Biswas et al., 2006). Table 2 below reports the mean VIQ and VC scores of the COS groups for each study with data from comparison groups, when applicable, as well as scales used (Table 2).

**Table 2:** Scores and measures of Verbal IQ (VIQ) or Verbal Comprehension Index (VC), characteristics for COS and comparative group.

Authors	COS								Comparison group(s)			Scale
	*M*	*SD*	*N*	Mean age of onset in yrs ±SD	Mean age in yrs ±SD	M/F ratio	Diagnostic tools	IQ exclusion criteria	*M*	*SD*	*N*	
Ayres and Heskett (1972)^a^	57 72^b^	NA	1	NA	6y11m	1F	NA	NA	NC	NC	NC	WISC^c^
Walker and Birch (1974)	82.60 80.05 77.20 77.15 80.45 84.60	11.06 14.30 9.06 8.44 12.99 16.01	20 20 20 20 20 20	NA	10 11 12 13 14 15	120M	NA	NA	NC	NC	NC	WISC^b^
Fish (1977)^a^	75^d^		1	3	10	1M	NA	NA	NC	NC	NC	WISC^b^ WAIS^b^
	87	NA			18							
	92		1	6	10	1F						
Inayatulla and Cantor (1980)^a^	52^e^ 58	NA	1	10	11y6m 11y10m	1M	DSM III	NA	NC	NC	NC	WISC-R
Carter et al. (1982)	87.7	19.53	15	NA	9.2	12/3	NA	NA	86.3^f^	16.52	18	WISC^b^
Caplan et al. (1990a)	88	16.31	31	NA	10.2 ± 1.5	25/6	DSM III	NA	NC	NC	NC	WISC-R
Caplan et al. (1992)	88	16.31	31	6.8 ± 1.98	10.3 ± 3.06	25/6	DSM III	NA	89^g^ 111^h^	24.13 20.37	27 58	WISC-R
Alaghband-Rad et al. (1995)	86.83^i^	29.1	7	Psychotic symptoms prior or by age 12	14.3 ± 1.8^j^	15/8^k^	DSM-III/-R	NA	NC	NC	NC	WISC^l^
Gartner et al. (1997)^a^	106^m^ 85 88	NA	1	NA	12	1M	DSM-IV	NA	NC	NC	NC	WISC-R WISC-III
Kumra et al. (1998)	89.19	15.68	15	10.31 ± 2.00	14.03 ± 2.16	14/15^n^	DSM-III-R	Premorbid IQ < 70	98.69^o^ 93.91^p^	12.31 9.82	19 14	WISC-R
Nicolson et al. (1999)	76.2^q^ 81.0	10.8 19.9	5 42	10.3 ± 1.8	14.3 ± 2.3	28/19	DSM-III-R	Premorbid IQ < 70	NC	NC	NC	WISC-III
Abu-Akel et al. (2000)	88.19^r^ 89.68^s^ 86.60^t^	15.68 17.26 14.23	32 17 15	NA	10.34 ± 1.56 10.37 ± 1.67 10.32 ± 1.48	27/5 13/4 14/1	DSM-III DSM-IV	NA	106.35^h^	14.90	34	WISC-R
Kumra et al. (2000)	91.2	13.0	17	10.7 ± 1.6	14.4 ± 1.8	12/5	DSM-III-R/DSM-IV	Premorbid IQ < 70	95.4^u^	12.0	21	WISC-R
Bailly and de Chouly de Lenclave (2004)^a^	74	NA	1	9	10	1F	NA	NA	NC	NC	NC	NA
Gochman et al. (2005)	92.7^v^ 83.1^w^ 75.6^x^ 81.1^y^ 79.7^z^ 78.5^aa^ 72.0^ab^	22.7 10.5 19.4 18.2 16.6 15.7 7.7	18 13 70 34 33 16 6	11.6 ± 1.6	8.6 ± 2.0 11.6 ± 1.6 14.3 ± 2.4 16.9 ± 2.6 18.7 ± 2.2 22.1 ± 2.8 24.3 ± 1.5	15/9 10/6 42/28 20/14 18/15 12/4 4/2	DSM-III-R/IV	IQ < 70	NC	NC	NC	WISC-R WISC-III
Biswas et al. (2006)	92.53	11.93	15	12.25 ± 1.16	18.52 ± 3.98	7/8	ICD-10 DCR	IQ < 70	103.45^ac^ 114.50^ad^	14.76 10.78	20 20	WAIS-R, MISIC
Stenstrøm et al. (2008)^a^	84	NA	1	NA	7	1 M	CIM10	NA	NC	NC	NC	WISC III
David et al. (2011)	87.8^ae^ 74.1^af^	20.17 15.1	24 94	10.7 ± 1.59 9.7 ± 2.1	15.1 ± 2.62^r^ 13.23 ± 2.79^s^	67/50	NA	IQ= or < 70	NC	NC	NC	NA
Ordóñez et al. (2016)	83.00^ag^ 75.58^ah^	15.97 15.10	53 38	9.51 ± 2.28^ai^ 10.29 ± 1.63	NA	53/38	DSM-III-R/DSM-IV	Premorbid IQ < 70	NC	NC	NC	WISC-R WISC-III WAIS-R WASI WASI II

NA: data not available; NC: no comparative group.

a Case study *N* = 1.

b Score at the end of the 6 months of treatment (tactile and vestibular stimulation on muscle tone).

c WISC or WAIS version not specified.

d Full scale IQ at 10 years old VIQ_10_ = 75; at 18 years old VIQ_18_ = 87 for same male patient; VIQ_10_ = 92 for female patient.

e Mean scores for same patient at two separate occasions.

f 18 patients hospitalized for psychiatric conditions other than schizophrenia: overanxious reaction of childhood (*N* = 10), unsocialized aggressive reaction of childhood or dyssocial behavior (*N* = 4), adjustment reaction of childhood or adolescence (*N* = 3), and depressive reaction of childhood (*N* = 1) (Carter et al., 1982).

g Mean score and standard deviation for complex partial seizures (epilepsy) patients.

h Mean score, standard deviation and N for the healthy control comparison group.

i Premorbid performance IQ.

j Mean age and ratio for *N* = 23, overall cohort, not only COS with available FSIQ data.

k Ratio for overall cohort (N = 23).

l WISC version not specified and only Verbal subscale mentioned.

m Patient's VIQ score in Middle of Grade 2 WISC-R, VIQ score in Fall of Grade 6 WISC-III and VIQ score in Spring of Grade 7, Shortly After Hospital Admission.

n Ratio for *N* = 29, overall cohort, not only COS who undergone VIQ testing.

o Mean score and standard deviation for the ADHD comparison group.

p Mean score and standard deviation for the Multidimensionally Impaired Disorder (MDI) comparison group.

q Mean score, standard deviation and N for COS with cytogenetic abnormalities.

r Mean score, standard deviation and ratio for all COS.

s Mean score, standard deviation and ratio for COS on Neuroleptics.

t Mean score, standard deviation and ratio for COS off Neuroleptics.

u Mean score and standard deviation for the Psychotic Disorder Not Otherwise Specified (PD-NOS) comparison group.

v Mean score and standard deviation of COS patients pre-onset.

w Mean score and standard deviation of COS patients post-onset.

x Mean score and standard deviation of COS patients at NIH admission.

y Mean score and standard deviation of COS patients at 2 years follow-up.

z Mean score and standard deviation of COS patients at 4 years follow-up.

aa Mean score and standard deviation of COS patients at 6 years follow-up.

ab Mean score and standard deviation of COS patients at 8+ years follow-up.

ac Mean score and standard deviation of AdOS: Subjects with adolescent schizophrenia.

ad Mean score and standard deviation of AOS: Adult schizophrenia subjects.

ae Mean score and standard deviation of COS patients without visual hallucination.

af Mean score and standard deviation for COS patients with visual hallucinations.

ag Mean score and standard deviation for male COS patients.

ah Mean score and standard deviation for female COS patients.

ai Mean age of onset for overall cohort (*N* = 72 males; *N* = 61 females), not only patients who undergone FSIQ testing.

Comparing COS group with less precocious groups affected by schizophrenia, Biswas et al. (2006) pointed out that the mean scores on the VIQ for COS patients are significantly lower than those of adolescent and adult schizophrenia groups. These statistical analyses reveal significantly different results *F*_(2)_ = 12.99, *p* < .001 between the three groups. On the other hand, when comparing male and female, males with COS (*N* = 72) have a significantly higher Verbal IQ compared to females (*p* = .03; Ordóñez et al., 2016). Looking for discrimination indicators in COS at WISC, Wechsler and Jaros (1965) found that scores (raw and standard) on the Comprehension and Similitudes subtests were higher than scores on the Arithmetic subtest, each by 3 points. Even if a more generalized state of emotional disturbance, rather than COS only, is being reflected in these signs (Kissel, 1966).

When comparing VIQ with an ASD group, no significant difference between COS and ASD children's scores were found and COS patients scored within the normal range on Vocabulary, Information, Comprehension, and Similarities subtests of the WISC-R (Asarnow et al., 1994). In addition, David et al. (2011) found that COS patients who experienced visual hallucinations had significantly lower Verbal IQ scores compared to those without visual hallucinations (*t*_(76)_ = 3.2, *p* = .002). The authors indicated that they observed a progressive decrease in Verbal IQ when considering the cumulative nature of hallucination modalities (visual, somatic, tactile and olfactory) in COS cohort. In other words, a greater number of hallucination modalities seems to be a factor in a lower VIQ mean score. Kumra et al. (2001b) found significant difference between COS patients and healthy controls in vocabulary score of WISC-R (*t*_(35)_ = 3.2, *p* = .003). Both COS and PD-NOS patients differed from healthy controls on this subtest.

#### 3.1.3 Visuo-spatial (VS) index, perceptual organization factor and performance IQ

Among studies, Performance IQ (PIQ) scores ranged on average from 53 to 105 with standard deviations from 5.0 to 20.6. These PIQ and Perceptual Organization Factor values are 1 to 2 standard deviations lower than normative data (Fish, 1977; Bailly and de Chouly de Lenclave, 2004; Nicolson et al., 1999; Ayres and Heskett, 1972; Walker and Birch, 1974, Ordóñez et al., 2016, etc.), although some studies show scores within the norms (Caplan et al., 1990a; Caplan et al., 1992; Alaghband-Rad et al., 1995; Biswas et al., 2006; Abu-Akel et al., 2000; Stenstrøm et al., 2008; Remschmidt, 2002). Table 3 below reports the mean scores of PIQ and the perceptual organization factor for COS and comparison groups, when applicable, according to the studies and the scale used (Table 3).

**Table 3:** Scores and measures of performance IQ or the perceptual organization index, characteristics for COS and comparative group.

Authors	COS								Comparison group(s)			Scale
	*M*	*SD*	*N*	Mean age of onset in yrs ±SD	Mean age in yrs ±SD	M/F ratio	Diagnostic tools	IQ exclusion criteria	*M*	*SD*	*N*	
Ayres and Heskett (1972)^a^	79^b^	NA	1	NA	6y11m	1F	NA	NA	NC	NC	NC	WISC^c^
Walker and Birch (1974)	90.25 85.25 84.40 78.60 87.10 87.00	13.07 14.60 15.26 18.48 20.38 17.10	20 20 20 20 20 20	NA	10 11 12 13 14 15	120 M	NA	NA	NC	NC	NC	WISC^c^
Fish (1977)^a^	61^d^		1	3	10	1M	NA	NA	NC	NC	NC	WISC^c^ WAIS^c^
	76	NA			18							
	78		1	6	10	1F						
Inayatulla and Cantor (1980)^a^	85^e^ 95 105	NA	1	10	10y2m 11y6m 11y10m	1M	DSM III	NA	NC	NC	NC	WISC-R
Carter et al. (1982)	82.3	13.72	15	NA	9.2	12/3	NA	NA	93.8^f^	17.73	18	WISC^c^
Caplan et al. (1990a)	93	16.27	31	NA	10.2 ± 1.5	25/6	DSM III	NA	NC	NC	NC	WISC-R
Caplan et al. (1992)	93	16.27	31	6.8 ± 1.98	10.3 ± 3.06	25/6	DSM III	NA	89^g^ 113^h^	20.61 14.99	27 58	WISC-R
Alaghband-Rad et al. (1995)	93.42^i^	19.0	7	Psychotic symptoms prior or by age 12	14.3 ± 1.8^j^	15/8^k^	DSM-III/-R	NA	NC	NC	NC	WISC^l^
Gartner et al. (1997)^a^	105^m^ 81 96	NA	1	NA	12	1M	DSM-IV	NA	NC	NC	NC	WISC-R WISC-III
Kumra et al. (1998)	85.28	16.80	15	10.31 ± 2.00	14.03 ±2.16	14/15^n^	DSM-III-R	Premorbid IQ < 70	99.58^o^ 90.86^p^	9.80 11.88	19 14	WISC-R
Nicolson et al. (1999)	67.0^q^ 75.9	5.0 20.6	5 42	10.3 ± 1.8	14.3 ± 2.3	28/19	DSM-III-R	Premorbid IQ < 70	NC	NC	NC	WISC-III
Abu-Akel et al. (2000)	93.33^r^	16.36	32	NA	10.34 ± 1.56	27/5	DSM-III DSM-IV	NA	105.66^h^	12.86	34	WISC-R
	93.86^s^	17.59	17		10.37 ± 1.67	13/4						
	92.80^t^	15.63	15		10.32 ± 1.48	14/1						
Kumra et al. (2000)	89.2	12.1	17	10.7 ± 1.6	14.4 ± 1.8	12/5	DSM-III-R/DSM-IV	Premorbid IQ < 70	91.5^u^	8.6	21	WISC-R
Bailly and de Chouly de Lenclave (2004)^a^	53	NA	1	9	10	1F	NA	NA	NC	NC	NC	NA
Gochman et al. (2005)	88.1^v^ 82.1^w^ 72.5^x^ 73.4^y^ 75.6^z^ 77.7^aa^ 72.4^ab^	17.9 14.8 18.6 18.6 17.8 13.8 11.9	18 13 70 34 33 16 6	11.6 ± 1.6	8.6 ± 2.0 11.6 ± 1.6 14.3 ± 2.4 16.9 ± 2.6 18.7 ± 2.2 22.1 ± 2.8 24.3 ± 1.5	15/9 10/6 42/28 20/14 18/15 12/4 4/2	DSM-III-R/IV	IQ < 70	NC	NC	NC	WISC-R WISC-III
Biswas et al. (2006)	83.73	11.13	15	12.25 ± 1.16	18.52 ± 3.98	7/8	ICD-10 DCR	<70	94.20^ac^ 104.35^ad^	13.05 9.62	20 20	WAIS-R, MISIC
Stenstrøm et al. (2008)^a^	129	NA	1	NA	7	1M	CIM10	NA	NC	NC	NC	WISC III
Ordóñez et al. (2016)	77.57^ae^ 77.05^af^	16.74 18.30	53 38	9.51 ± 2.28^ag^ 10.29 ± 1.63	NA	53/38	DSM-III-R/DSM-IV	Premorbid IQ < 70	NC	NC	NC	WISC-R WISC-III WAIS-R WASI WASI II

a Case study *N* = 1.

b Score at the end of the 6 months of treatment (tactile and vestibular stimulation on muscle tone). At baseline, no performance score could be obtained.

c WISC or WAIS version not specified.

d Full scale IQ at 10 years old PIQ_10_ = 61; at 18 years old PIQ_18_ = 76 for same male patient; PIQ_10_ = 78 for female patient.

e Mean scores for same patient at three separate occasions.

f 18 patients hospitalized for psychiatric conditions other than schizophrenia: overanxious reaction of childhood (*N* = 10), unsocialized aggressive reaction of childhood or dyssocial behavior (*N* = 4), adjustment reaction of childhood or adolescence (*N* = 3), and depressive reaction of childhood (N = 1) (Carter et al., 1982).

g Mean score and standard deviation for complex partial seizures (epilepsy) patients.

h Mean score, standard deviation and N for the healthy control comparison group.

i Premorbid performance IQ.

j Mean age and ratio for *N* = 23, overall cohort, not only COS with available FSIQ data.

k Ratio for overall cohort (N = 23).

l WISC version not specified and only Verbal subscale mentioned.

m Patient's PIQ score in Middle of Grade 2 WISC-R, PIQ score in Fall of Grade 6 WISC-III and PIQ score in Spring of Grade 7, Shortly After Hospital Admission.

n Ratio for *N* = 29, overall cohort, not only COS who undergone VIQ testing.

o Mean score and standard deviation for the ADHD comparison group.

p Mean score and standard deviation for the Multidimensionally Impaired Disorder (MDI) comparison group.

q Mean score, standard deviation and N for COS with cytogenetic abnormalities.

r Mean score, standard deviation and ratio for all COS.

s Mean score, standard deviation and ratio for COS on Neuroleptics.

t Mean score, standard deviation and ratio for COS off Neuroleptics.

u Mean score and standard deviation for the Psychotic Disorder Not Otherwise Specified (PD-NOS) comparison group.

v Mean score and standard deviation of COS patients pre-onset.

w Mean score and standard deviation of COS patients post-onset.

x Mean score and standard deviation of COS patients at NIH admission.

y Mean score and standard deviation of COS patients at 2 years follow-up.

z Mean score and standard deviation of COS patients at 4 years follow-up.

aa Mean score and standard deviation of COS patients at 6 years follow-up.

ab Mean score and standard deviation of COS patients at 8+ years follow-up.

ac Mean score and standard deviation of AdOS: Subjects with adolescent schizophrenia.

ad Mean score and standard deviation of AOS: Adult schizophrenia subjects.

ae Mean score and standard deviation for male COS patients.

af Mean score and standard deviation for female COS patients.

ag Mean age of onset for overall cohort (*N* = 72 males; *N* = 61 females), not only patients who undergone FSIQ testing.

In a longitudinal study, patients showed significant drop in PIQ (*p* = .05) between an average of 2 years before the onset of the disease and 1.7 years afterwards, with an average loss of 6.08 points (Gochman et al., 2005). For their part, Asarnow et al. (1994) found that COS children obtained scores within the norm on the perceptual organization factor of the WISC-R. Looking for indicators of differentiation between COS profiles from non-COS profiles, Wechsler and Jaros (1965) found that scores on Image Complements subtest being 3 points higher than Image Arrangement subtest, as well as on the Object Assembly subtest compared to the Code subtest. According to the authors, high variability between these subtests seems to be most effective feature for distinguishing COS. In addition, a cut-off score of ±16 points between verbal and performance scale (V—P) proved to be a significant and optimal discriminant and was added as another possible diagnostic signs of COS. Kissel (1966) highlighted that not only COS population responded to these identified signs, contradicting Wechsler and Jaros.

Alternatively, Walker and Birch (1974) studied PIQ and VIQ tendencies in COS patients and suggested that, at every age level, PIQ was higher than VIQ, and, for all ages combined, mean Verbal IQ was significantly lower (*t* = 4.81, *p* < .01). Moreover, from FSIQ of 74–75, this discrepancy is reversed where lower-IQ COS children demonstrated greater competence with Verbal subtests, but as IQ rose above 75, Performance IQ was increasingly superior to Verbal IQ.

Concerning comparisons of PIQ with other groups, mean scores for perceptual organization does not differ significantly between COS and ASD children (Asarnow et al., 1994). COS patients show lower PIQ scores compared to adolescent and adult groups (*F*_(2)_ = 14.15, *p* = .001; Biswas et al., 2006). Furthermore, comparing COS, ADHD and Multidimensionally Impaired Disorder (MDI) groups, there is a significant difference between scores of the three groups on their perceptual organization sub-score (*F* = 7.26, *p* < .01) with COS showing lower scores than the two other groups (Kumra et al., 1998). Furthermore, when COS patients are divided in two groups, patients with cytogenetic abnormalities had lower PIQs *p* = .04 (but not verbal or FSIQs) compared to those without such anomalies (Nicolson et al., 1999).

Kumra et al. (2000) found a non-significant difference between means of COS and PD-NOS groups in perceptual organization factor scores (*t* = 0.68, *p* = .5) of WISC-R. According to Ordóñez et al. (2016), no significant difference was observed between mean score of men and that of women in PIQ (*t*_89_ = 0.14, *p* = .89). Carter, Alpert and Stewart showed significantly lower scores for COS in WISC performance measure compared to “nonschizophrenic” comparison group (*p* < .05; Carter et al., 1982). Using WISC-R and other tools, such as Bender Gestalt, Beery's Test of Visual Motor Integration, COS cohort showed common visual-spatial deficits (McKenna et al., 1994). Similarly, significant difference between COS patients (*M* = 7.3 ± 3.8) and healthy controls (*M* = 12.1 ± 2.9) in block design score of WISC-R *t*_(38)_ = 3.8, *p* = .001 was found. Both COS and PD-NOS patients differed from healthy controls in this subtest (Kumra et al., 2001b).

#### 3.1.4 Fluid reasoning (FR), working memory (WM) and processing speed (PS) indexes

To our knowledge, there is no detailed data available in the literature using the WISC-IV and WISC-V indicating Fluid Reasoning (FR) Index, Working Memory (WM) Index and Processing Speed (PS) Index scores in COS. The results of all attention tests, which have been used to measure processing speed in COS, are presented in the section on processing speed of attentional processes.

#### 3.1.5 Distractibility factor of WISC-R

A measure of processing speed was previously presented as “distractibility factor” in WISC-R version. Remschmidt (2002) noted specific cognitive deficit in distractibility factor for COS. Abu-Akel et al. (2000) suggested that speech functions in COS, characterized by the use of inappropriate and implied (indirect) responses to questions, may be reflected by distractibility, rather than global intellectual impairments. Correlation observed between loose associations, also linked to the WISC-R distractibility factor, and both inappropriate and implied responses further emphasizes the potential relationship between cognitive deficits and the impaired communication in COS children. Finally, when comparing with an ASD group, COS cohort showed significantly lower scores for COS than ASD children on WISC-R' distractibility factor. COS children's scores were more than one standard deviation below the mean and, importantly, significantly lower than scores they obtained on the verbal comprehension and perceptual organization factors (Asarnow et al., 1994). In his review, Caplan (1994) also reported lower WISC-R distractibility factor for COS compared to ASD children. Measuring processing speed with WISC-R' subtests, subjects with COS exhibited cognitive deficits in Symbols subtest in visuomotor modality (Kumra et al., 2000).

#### 3.1.6 IQ decline and link with symptoms

A significant decline in IQ was observed by Bedwell et al. (1999), who noted a significant drop in “post-psychotic” FSIQ scores with a mean slope of 0.21, *SD* = 0.40 (*Wilcoxon T* = 115, *N* = 36, *p* = .001). Three of the 11 subtests assessed as a function of age decreased significantly, notably image arrangement (*Wilcoxon T* = 58, *N* = 28, *p* = .003), information (*Wilcoxon T* = 93, *N* = 30, *p* = .02), and block design (*Wilcoxon T* = 83, *N* = 29, *p* = .01). The authors' explanatory hypotheses for this ‘post-psychotic’ decline are scoring of IQ during the school years, greater progression of cerebral abnormalities observed in COS cases and the greater severity of disorders in these young patients compared with adults. This decline did not indicate a deterioration but rather a difficulty in acquiring new knowledge, abilities and problem-solving skills compared to healthy control. However, the group showed no absolute decline in any of the raw scores observed. Information and Comprehension subtests of the WISC-R and WISC-III, were close to zero and showed no change. For Information, mean slope was equal to 0.03, *SD* = 0.10, range of values between 0.22 and 0.30 (*Wilcoxon T* = 245, *N* = 31, *p* = .18); for Comprehension, mean slope was equal to 0.05, SD = 0.21, range of values between 0.31 and 0.67 (*Wilcoxon T* = 223, *N* = 30, *p* = .42). In their review, Jacobsen and Rapoport (1998) also noted a significant deterioration in intellectual functioning between the premorbid period and after psychosis onset (*p* = .002) that continue to decline 24 to 48 months after the onset of psychosis (*p* < .05).

During a prospective longitudinal study measuring changes in IQ for subjects in premorbid phase, from 2 years before the onset of COS (*N* = 18) to 8 years after its onset (*N* = 6). Gochman et al. (2005) reported that mean FSIQ scores before disease onset (available for 18 subjects) went from average (*M* = 90.0; *SD* = 20.0) to low or borderline average after COS onset (*M* = 80.0; *SD* = 11.1) between, on average, 2 years before disease onset and 1.7 years afterwards. Authors also observed a significant decline in VIQ (Verbal Comprehension; *p* = .02) and VS (or visuo-spatial index; *p* = .05) with an average loss of 9.59 and 6.08 points, respectively. On the other hand, researchers found no further deterioration in overall IQ during the follow-up period of 2 to 8 years and up to >13 years after the onset of the condition. However, Alaghband-Rad et al. (1995) did not reveal consistent cognitive decline between pre- and postpsychotic IQ testing (Prepsychotic *M* = 87.7; *SD* = 25.4, postpsychotic *M* = 83.7; *SD* = 17.3, *paired t* = 0.77, *p* = .48). Similarly, Goldfarb et al. (1969) stipulated that, during residential treatment, COS patients with FSIQs of 60 and over show an improvement in FSIQ scores over a three-year period (the rank coefficient of correlation being 0.03).

Regarding relationship to symptoms, among 125 COS, patients with mixed profile of positive and negative symptoms had the worst cognitive deficits. FSIQ differed between three symptoms groups (*F*_(2,111)_ = 6.59, *p* = 1.97E-03), as low mixed (low scores on both dimensions of SAPS/SANS; Andreasen et al., 1995) had a significantly higher score than high mixed (high scores on both dimensions; *p* = 1.50E-03). According to the authors, the functional impact in patients with mixed and low-intensity symptoms is more closely related to psychotic symptomatology and cognitive impairments than by externalizing symptoms or prominent comorbidities (Craddock et al., 2018). Visual hallucinations are also significantly associated with a lower IQ score (*p* < .01; David et al., 2011). Remschmidt (2002) found a correlation between symptomatology and IQ, where COS patients with high IQ showed more positive and less negative symptoms than those with low IQ. Looking at illogical thinking, epileptic children had a significantly higher mean score than COS children only at a FSIQ score of 85 (*z* = 7.33, *p* < .001). For all IQ levels, the estimated mean illogical thinking scores were significantly higher for schizophrenic than healthy controls children (*z*_(85)_ = 1.98, *p* < .05; *z*_(100)_ = 2.98, *p* < .01; *z*_(115)_ = 3.90, *p* < .001; Caplan et al., 1992).

On the other hand, in their 2001 study, Thompson et al. (2001) showed that COS patients with the most global tissue deficits (measured by Magnetic Resonance Imaging; MRI) had the lowest IQs at follow-up (*r* = 0.62; *p* < .016). The amount of grey matter on the initial scan was also a good predictor of FSIQ in the patient group at follow-up (*r* = 0.52; *p* < .042). Similarly, COS patients showing positive neurological findings have lower FSIQ scores than patients without neurological findings (nonorganic; Goldfarb et al., 1969).

### 3.2 Neuropsychological substrate of attention in COS

#### 3.2.1 Sustained and selective attention

Zahn et al. (1998) used evaluated reaction time as a measure of sustained attention and showed that reaction time was slower in COS patients than their control group with significantly longer reaction time in all conditions for COS patients. Authors suggested that these deficits are greater as the number of operations required to obtain a response increases with errors being impulsive in nature rather than random loss of attention. Unlike controls, whose reaction times for errors were longer and showed higher variance -likely due to loss of attention or attempts to correct errors- COS patients showed impulsive errors possibly unrecognized and reaction time for errors in was almost at the level of their usual series. Finally, authors indicated that a slow reaction time is considered a non-specific marker of generalized dysfunction.

Using the Continuous Performance Task as a measure of sustained attention (CPT; Halperin et al., 1991), Strandburg et al. (1990) found that COS children made more errors of omission and commission than controls. The difference in overall performance between groups was significant (*F*_(30)_ = 22.7, *p* < .0001) and the average percentage of success was 86.6 % ± 9.6 for controls (*N* = 19) and 66.9 % ± 11.9 for COS patients (*N* = 13). Authors suggested that impaired selective attention might be a factor leading to a “defective filter” in COS. Thus, the addition of distractor stimuli to the CPT task, placing additional demands on selective attention, appears to reduce the performance of COS patients largely compared to controls. Through three experiments, Asarnow et al. (1986), showed impairment in controlled attentional processes for COS patients while more automatic modes of attending are relatively intact. According to the authors, this impairment in information processing would originate in their inability to regulate mobilization and direction of attention, and to discriminate target stimuli. Similarly, COS showed impaired performance compared to matched controls (*F*_(2,35)_ = 8.39, *p* < .001) which suggested an information-processing impairment in COS patients was independent of general level of intellectual skills, as measured by PPVT mental age. These deficits could not be accountable to iconic or immediate memory. COS subjects seemed to use the same information acquisition strategy as that employed by the older matched healthy comparison group but less efficiently (Asarnow and Sherman, 1984).

Kumra et al. (2001b) compared smooth pursuit eye tracking of COS and PD-NOS patients compared to healthy comparison group. They found that subjects with COS (79.3 %, *N* = 23 of 29) showed significantly more eye-tracking dysfunction than controls (20.0 %, *N* = 4 of 20) (χ^2^ = 14.3, *df* = 1, *p* < .01). COS subjects had qualitatively poorer eye tracking, higher root mean square error, lower gain, and a greater frequency of catch-up saccades than healthy volunteers. They concluded that both COS and PD-NOS patients differed from healthy children in attentional disturbances.

#### 3.2.2 Processing speed

Strandburg et al. (1994) used the Span of Apprehension task performance (Asarnow and MacCrimmon, 1981), to measure the efficiency of visual information processing where control children performed significantly better than COS children (total correct: control = 82 %, COS = 70 %, *p* < .001). Authors argued that the observed deficits in Span performance in COS subjects resulted from deficits in the allocation of resources to controlled attentional processes, most likely those associated with serial search for the visual icon. Similarly, COS patients showed impaired performance on the partial-report version of the span of apprehension compared to a control group matched on general intellectual abilities (Asarnow and Sherman, 1984). Using the partial report span of apprehension task, Caplan (1994) found that information-processing deficits primarily emerged during controlled processing rather than automatic processing.

Caplan et al., 1990a, Caplan et al., 1990b hypothesized that loose association and illogical thinking might represent different aspects of attention processing in patients with COS, illustrated by deficits in the speed of visual information processing. Illogical thinking seems to be linked to a deficit in momentary processing capacity, measured by the Apprehension Span Task. Disorganized speech, on the other hand, seems to reflect patients' ‘distractibility’, as indicated by a negative correlation with the score of the distractibility factor of the WISC-R. Processing speed was measured using the Trail Making Test A and B in visuomotor modality (TMT A and B; Sánchez-Cubillo et al., 2009) where patients with COS exhibited cognitive deficits (Kumra et al., 2000). Moreover, visual information processing measured using the Span of Apprehension tasks demonstrated that children with COS have delayed initiation of serial search and/or carry out serial search more slowly than controls. COS patients show significant deficit in attentional processes and allocation of attentional resources necessary for efficient and accurate processing (Jacobsen and Rapoport, 1998). Remschmidt (2002) indicated that attentional deficit at an early age may be primary and an important link to later social dysfunctioning in schizophrenia. The relationship between attentional dysfunction and social skills became significant at mid-adolescence. Also, these attentional deficits seemed to be a stable trait throughout the development in subjects at risk for schizophrenia. Measuring reaction time, Czudner and Marshall (1967) showed that COS children are generally slower and more variable than control or cultural-familial children in reacting to a visual stimulus.

### 3.3 Neuropsychological substrate of memory

#### 3.3.1 Working memory

Loeb et al. (2018) examined the neural correlates of WM in COS patients compared to their siblings and controls using the N-back task (3 sequences 0, 1, and 2-back; Owen et al., 2005). Firstly, only 32 out of the 55 selected participants were able to perform the task, thus participated in the final experiment. Their results showed significantly lower accuracy scores in both identity and location conditions, for both 1 and 2-back sequences, compared to controls (*Cohen's d* = 1.0, *p* < .001 for 1-back; *Cohen's d* = 1.3, *p* < .001 for 2-back). Moreover, more than half of the COS patients who were able to perform the 1-back tasks were unable to perform the 2-back tasks. As for the siblings, they did not obtain significantly lower accuracy scores than the controls for any task. However, their accuracy was generally lower than that of controls, and effect sizes increased as the workload increased from one to 2-back with *Cohen's d* = 0.1, *p* = .945 for 1-back; *Cohen's d* = 0.5, *p* = .161 for 2-back.

Karatekin and Asarnow (1998) found that COS patients exhibited deficits in both verbal and spatial working memory. COS children showed deficits in Digit Span, indicating deficient verbal WM. Group effect analyses, with data grouped according to order of digit recall, indicated that the control group remembered more digits than COS group, (*t*_35_ = 3.29, *p* = .002; *effect size* = 1.05). Regarding spatial WM, COS group was not significantly different from control in the immediate recall condition of the Dot Test. However, they exhibited an impairment in delayed recall condition (*M* = 32.4, *SD* = 11.5) with a higher distance error rate compared to the control group (*M* = 23.1, *SD* = 11.2; *t*_38_ = 2.44, *p* = .019). These results are consistent with those of White et al. (2010), where COS patients performed worse than control on both verbal and visuospatial tasks of the Sternberg Item Recognition Paradigm (SIRP; Sternberg, 1969).

#### 3.3.2 Other memory function processes

Regarding overall memory processes, Kumra et al. (2000), showed significant deficits in COS patients in verbal learning, visual learning, and global memory compared to a control group. These results aligned with those obtained by Biswas et al. (2006) where COS patients had markedly below-average scores for measurement of global memory and the subtests of mental balance, verbal retention of dissimilar pairs, and recognition.

COS children obtained significantly lower results compared to matched control subjects when adding tasks involving short-term memory (Visual Retention Test; Benton, 1974, and Visual Form Discrimination Test; Benton et al., 1977). COS subjects also exhibited pathological results in the complex analytical test of block design (recalling shapes), as well as significantly lower results compared to matched control subjects when solving multi-step problems on the Block Design task that required retaining and updating, sub-goals within a sequential problem-solving approach (Asarnow et al., 1994). Using a modified version of Wisconsin General Test Apparatus (WGTA; House and Zeaman, 1958) to test visual discrimination task based on form, color and size, Ottinger et al. (1965) showed a significant decrease in errors over trials in COS patients which is interpreted by the authors as learning on each task. Nevertheless, COS performance was lower than controls.

Finally, COS children show more deficit than PD-NOS on the verbal learning subtest (Wide Range of Memory and Learning, WRAML; Sheslow and Adams, 2003; Kumra et al., 2000). Interesting finding of a deficit in verbal learning in COS patients not responsive to neuroleptics, as several studies have shown that COS patients have a selective deficit in learning (Palmer et al., 1997; Saykin et al., 1994), and deficits in verbal learning are associated with functional impairments (Green, 1996; Kumra et al., 2000).

### 3.4 Neuropsychological substrate of executive function

#### 3.4.1 Flexibility

Using the Wisconsin Card Sorting Test (WCST; Heaton, 1981) COS patients gave significantly more perseverative responses (sorting according to a rule that is no longer correct or a rule that systematically leads to an incorrect response) than controls. The implementation of a sorting strategy did not improve their performance. On the contrary, their performance significantly deteriorated after this instruction. COS group significantly increased the number of non-perseverative errors (random responses) between the first and second half of the WCST, whereas ASD children and control children showed no significant change (Asarnow et al., 1994). Similarly, COS children showed a deficient percentage of perseverative errors on the WCST (*M* = 80.4 %, *SD* = 14.0; Kumra et al., 2000; Malhotra et al., 2006). These cognitive impairments on tasks such as the WCST are shown to be analogous to those observed in adults diagnosed with schizophrenia later in life (Kumra et al., 2001a).

#### 3.4.2 Planification

COS subjects exhibited deficits in the visuospatial planning task of the Rey-Osterrieth Complex Figure (Rey-Osterrieth Complex Figure; Berry et al., 1991), showing impaired planification (*M* = 57.0, *SD* = 38.3; Kumra et al., 2000). Considering main executive functions, COS patients show generalized cognitive deficits (Kumra et al., 2000, Kumra et al., 2001a; Asarnow et al., 1994). To our knowledge, no other executive function measures and results are available for COS patients.

### 3.5 Verbal and learning disabilities

Even though it is not our subject of study, other types of disorders can be observed in patients with COS, notably language disorders and learning disorders. A significant difference between the Full Scale WISC IQ scores (*M* = 84.7; *SD* = 16.2) and the average standard scores of COS for both the spelling subtests (*M* = 97.7; *SD* = 16.1, *N* = 23, *t* = 4.0, *p* = .001) and reading decoding subtest (*M* = 97.7; *SD* = 13.7, *N* = 23, *t* = 3.7, *p* = .001) of the Kaufman Test of Educational Achievement (KTEA; Kaufman and Kaufman, 1985) is observed. Coulon et al. (2020) showed that COS patients had more history of learning disorders than the AOS group (25 % for COS versus 9.2 % in adults; *p* = .020).

Regarding decoding and reading comprehension, in a study comparing of COS, ADHD, and Multidimensionally Impaired Disorder (MDI) groups during tasks, MDI and COS children experienced considerable difficulty understanding what they were reading despite adequate word recognition skills. Reading decoding skills were significantly better than reading comprehension skills (*p* < .05; Kumra et al., 1998). Among 88 cases of SSD, more learning disorders or developmental delays in COS subjects are observed. They also received more diagnoses of mild intellectual disability (moderate/severe intellectual disability being excluded from the study). In this cohort, comprising 66 (75 %) COS cases, 14 (16 %) presented specific verbal learning disorders, 30 (34 %) specific non-verbal learning disorders, and 36 (41 %) any specific learning disorder (Giannitelli et al., 2020).

In their review, Courvoisie et al. (2001) reported that COS children exhibited a notable prevalence of language disorders, encompassing expressive, receptive, and specific impairments. These deficits directly contributed to thought disorder and disorganization. Although children experiencing delayed expressive and receptive language development may eventually catch up with their peers, they often retain deficiencies in their linguistic abilities. Individuals with COS who exhibited premorbid speech and language impairments demonstrates a higher familial predisposition to schizophrenia spectrum disorders and experienced more obstetrical complications. According to the authors, these findings indicated that the pathophysiology of schizophrenia entails abnormal development of language-related functions.

Finally, using verbal and imagery tasks such as the Word Association Task (Ervin, 1961) and the Primary Mental Abilities-Verbal Meaning Test (Thurstone, 1963), Carter et al. (1982) showed that when presented with nonverbal stimuli, both COS and control subjects performed similarly. However, when the stimulus is verbal, COS patients performed less effectively compared to control subjects, with results approaching statistical significance. COS subjects demonstrated performance more akin to non-schizophrenic subjects when they can utilize cues from both verbal and nonverbal modalities, rather than relying solely on verbal cues.

#### 3.5.1 Peabody Picture Vocabulary Test as a measure of verbal intelligence

The Peabody Picture Vocabulary Test (PPVT; Dunn, 1959; Dunn and Dunn, 1981; Dunn and Dunn, 2007) may be used to measure verbal intelligence. Karatekin and Asarnow (1998) reported below-average level in COS patients. PPVT-R test seemed less sensitive than Wechsler scales to test-retest effects, which were present in some of the children. The PPVT-R scores in the control and ADHD groups were almost identical and about one standard deviation higher than the scores of COS. The latter had significantly lower scores than control children (*t*_(37)_ = 2.48, *p* = .018) and ADHD children (*t*_(41)_ = 2.80, *p* = .008), who did not differ between them. Respectively, *M* = 103.2, *SD* = 16.3 for controls, *M* = 104.5, *SD* = 17.4 for ADHD group and *M* = 89.1, *SD* = 16.6 for COS group.

Nevertheless, Asarnow et al. (1994) showed, in a meta-analysis of the studies carried out in their laboratory, that scores of children with COS were not significantly different from those of control children on the PPVT-R (*M* = 93.9, *SD* = 14.7 for COS group). Same observation for Waterhouse and Fein (1984) who did not find any significant differences in the PPVT between COS, ASD and another group called NANS (“neither Rutter Autistic nor Creak Schizophrenic”). A one-way analysis of variance (ASD, *N* = 107, *M* = 31.55, *SD* = 25.32; COS, *N* = 82, *M* = 37.12, *SD* = 22.70; NANS, *N* = 47, *M* = 38.56, *SD* = 24.13) gave a *F*_(2,233)_ = 1.76, which was not significant. The mean PPVT for the sample of 203 control children was *M* = 104.52, *SD* = 13.

## 4 Discussion

The 66 selected articles focus on neurocognitive impairments in patients with Childhood-Onset Schizophrenia (COS). These children show neurocognitive dysfunction in intelligence measured by Full Scale Intelligent Quotient (FSIQ)'s mean scores ranging from one to two standard deviation lower than normative data on WISC scales (Caplan et al., 1992; Kumra et al., 2000; Kumra et al., 2001a; Biswas et al., 2006, Fernandez et al., 2021). These deviations from norms are also found for Verbal IQ (VIQ) and Performance IQ (PIQ) scores (Nicolson et al., 1999; Ordóñez et al., 2016). Children afflicted with COS show significantly more impairment than adolescent does and adult forms (Biswas et al., 2006). These children display significant deterioration in intellectual functioning between premorbid period and after onset of psychosis (Bedwell et al., 1999; Gochman et al., 2005). Severity of symptoms (hallucination cumulative modalities, David et al., 2011; high scores on both dimensions of SAPS/SANS, Andreasen et al., 1995; and neurological deficits, Thompson et al., 2001) is associated with lower IQ in COS patients.

Similarly, this review highlights slower reaction time (Zahn et al., 1998) and more errors of omission and commission than controls in COS patients (Strandburg et al., 1990). Patients showed impairment in sustained and selective attention that could be due to impulsive errors or an inability to regulate mobilization and direction of attention and seem to be independent of general level of intellectual skills (Asarnow and Sherman, 1984; Asarnow et al., 1986; Zahn et al., 1998). These attentional deficits are also observed in processing speed (Kumra et al., 2000) as a difficulty in allocation of resources to controlled attentional processes (Strandburg et al., 1994; Jacobsen and Rapoport, 1998). Impairments in memory processes were noticed mainly in working memory for verbal and spatial modalities (Karatekin and Asarnow, 1998; White et al., 2010; Loeb et al., 2018) as well as verbal, visual and visuospatial learning (Asarnow et al., 1994; Saykin et al., 1994; Palmer et al., 1997; Kumra et al., 2000). COS patients presented cognitive impairments on tasks measuring flexibility (Asarnow et al., 1994; Kumra et al., 2000; Kumra et al., 2001a; Malhotra et al., 2006) and planification in visuospatial modality (Kumra et al., 2000) as processes of executive function. Finally, language and learning dysfunctions were shown in COS patients (Carter et al., 1982; Kumra et al., 1998; Courvoisie et al., 2001; Giannitelli et al., 2020) and PPVT-R's reported scores were below-average level in COS patients (Karatekin and Asarnow, 1998).

These results are consistent with impairment found in Early-Onset Schizophrenia (EOS) (Frangou, 2013; Pontillo et al., 2021), and Adult-Onset Schizophrenia (AOS) (Abramovitch et al., 2021; Fusar-Poli et al., 2012; Guo et al., 2019; Heinrichs and Zakzanis, 1998; Keefe et al., 2011; Nuechterlein et al., 2004; Mesholam-Gately et al., 2009).

Neurocognitive impairments highlighted do not appear to be specific to COS but are more likely to be transdiagnostic within mental illnesses (Hilton et al., 2024) and, in the field of schizophrenia, argue in favor of the well-admitted hypothesis of a continuum between COS and AOS (Driver et al., 2020).

Similar cognitive impairments also appear in early-onset psychosis (COP; Hagen et al., 1968; Brodsky et al., 2014), broader spectrum than COS, as well as in some genetic syndromes, such as 22q11 deletion syndrome, where children display deficits in intelligence, sustained attention, executive functioning, and verbal working memory compared to controls (Lewandowski et al., 2007; Sato et al., 2020), and in patients with variants (or mutations) of schizophrenia risk alleles (Riglin et al., 2017), and lastly in children at risk of developing schizophenia (patients' offspring suffering from schizophrenia's; Chapman, 1979; Ross et al., 2006).

The heterogeneity in diagnostic measures and scales, stemming from different versions of DSM and ICD versions, as well as differences in assessment tools, or a lack of phenotype data in studies complicate participants' categorization. The variability in intelligence measurements and scales, including versions of the WISC and WAIS, poses the same challenge in comparing cognitive functioning across studies. The use of different exclusion/inclusion thresholds, such as varying IQ criteria, may influence the composition of study samples and thus affect observations. The diverse clinical profiles of COS patients, including differences in symptomatology, comorbidities, age of onset, treatment status and disparities in testing conditions (from hospitalization settings to academic environments), introduce complexity and potential confounding that may affect the interpretation of results.

Finally, the clinical heterogeneity of COS, as well as the variability of the conditions under which cognitive impairment was measured, meant that it was not possible in this study to determine a specific pattern of impairment, but rather an overall and unspecific impairment. However, the severity of cognitive dysfunction in COS, combined with the diagnostic challenge facing clinicians, advocates better neuropsychological characterization of these patients from the initial phases of the disease and repeatedly over time. A family approach is also recommended insofar as a growing body of literature also demonstrates a familial transmission of neurocognitive phenotypes, with parents and siblings of COS patients also showing significantly different and impaired performance from controls (Gochman et al., 2004; Bigdeli et al., 2020; Asarnow et al., 2002; Wagshal et al., 2012, Wagshal et al., 2015; Alaghband-Rad et al., 1995; Kumra et al., 2001a; Havelkova, 1967).

### 4.1 Limitations

We have focused on neuropsychological function including intelligence, attention, memory and executive functions because one of our goals is to offer insights on neurocognitive dysfunction for future target of early intervention programs in cognitive remediation for patient suffering from COS. Thus, we have excluded language and motor dysfunctions.

Our reliance on a single database may restrict the diversity and scope of our article's sample that is why we choose to have no restriction of date and language.

A meta-analysis would allow us to better explore the influence of the neuropsychological test used and the version of the DSM diagnostic classification on the nature of the results highlighted in these studies.

### 4.2 Perspectives

As evaluation of neuropsychological characteristics drive treatment and cognitive remediation, future research should detail more their data (e.g., all indexes of WISC scales not limited to arbitrary threshold and more in-depth phenotypic data), and focus on understanding the developmental course of cognition (including social cognition) with both categorical and dimensional approaches. A multimodal approach would also be recommended to fully grasp global vision of COS. This approach would ideally include clinical, neuropsychological, neuroimaging and genetics perspectives (Ordóñez et al., 2015).

## 5 Conclusion

The present review, therefore, contributes to a growing body of evidence suggesting that COS is characterized, as AOS and other neuropsychiatric conditions, by major and unspecific neurocognitive impairments. Their rigorous and systematic assessment remains essential in COS, as they are predictive factors of a worse prognosis than in AOS, and a better understanding of these impairments will pave the way for more precise and targeted intervention.

## CRediT authorship contribution statement

**A. Armita:** Writing – review & editing, Writing – original draft, Visualization, Validation, Supervision, Project administration, Methodology, Investigation, Formal analysis, Data curation, Conceptualization. **J. Guivarch:** Writing – review & editing, Visualization, Validation, Supervision, Project administration, Conceptualization. **E. Dor:** Writing – review & editing, Writing – original draft, Visualization, Validation, Supervision, Methodology, Investigation. **G. Laure:** Writing – review & editing, Validation, Supervision, Project administration, Methodology, Investigation, Formal analysis, Data curation, Conceptualization. **R. Zeghari:** Writing – review & editing, Visualization, Validation, Formal analysis. **M. Gindt:** Writing – review & editing, Visualization, Validation, Supervision, Formal analysis. **S. Thümmler:** Writing – review & editing, Validation, Supervision, Methodology. **F. Askenazy:** Writing – review & editing, Project administration, Methodology, Investigation, Formal analysis, Data curation, Conceptualization. **A. Fernandez:** Writing – review & editing, Writing – original draft, Visualization, Validation, Supervision, Resources, Project administration, Methodology, Investigation, Formal analysis, Data curation, Conceptualization.

## Funding sources

The authors declare no funding sources.

## Declaration of competing interest

The authors declare that they have no known competing financial interests or personal relationships that could have appeared to influence the work reported in this paper.
